# Preventive Effects of Tryptophan–Methionine Dipeptide on Neural Inflammation and Alzheimer’s Pathology

**DOI:** 10.3390/ijms20133206

**Published:** 2019-06-29

**Authors:** Yasuhisa Ano, Yuka Yoshino, Kazuyuki Uchida, Hiroyuki Nakayama

**Affiliations:** 1 Graduate School of Agricultural and Life Sciences, the University of Tokyo, Tokyo 113-8657, Japan; 2 Research Laboratories for Health Science & Food Technologies, Kirin Holdings Co. Ltd., Kanagawa 236-0004, Japan

**Keywords:** Alzheimer’s disease, amyloid β, cognitive function, dipeptide, inflammation, microglia

## Abstract

Preventive approaches for age-related memory decline and dementia have become a high priority in the aging society because of the lack of therapeutic approaches. Recent epidemiological studies have reported that fermented dairy products can help prevent dementia. Previously, we identified tryptophan–tyrosine (WY) and tryptophan–methionine (WM) peptides as the suppressants of activation of the primary microglia and showed that WY peptide consumption suppresses inflammation in the brains of Alzheimer’s disease model mice. However, the effects of the WM peptide on inflammation in the brain and Alzheimer’s pathology have not been investigated. Here, we evaluated the effect of WM peptide consumption on Alzheimer’s disease model (5×FAD) mice. In 5×FAD mice, intake of WM peptide suppressed the production of inflammatory cytokines, activation of microglia, and infiltration of activated microglia around β amyloid (Aβ) depositions. WM peptide intake reduced Aβ deposition in the cortex and hippocampus and then improved the object recognition memory. Taken together with previous reports, the current findings indicate that ingestion of tryptophan-related peptides or food material rich in tryptophan-related peptides, thereby regulating microglial activity, represents a potential preventive approach for cognitive decline and dementia related to inflammation.

## 1. Introduction

With the rapid growth of aging populations worldwide, cognitive decline and dementia have become an increasing burden for society. Because of the absence of an effective therapeutic approach for dementia, preventive approaches are receiving increasing attention. Approximately 60% of dementia is classified as Alzheimer-type dementia. In Alzheimer’s disease (AD), β amyloids (Aβs) and phosphorylated tau become aggregated and are deposited as a senile plaque and a neurofibrillary tangle (NFT) [1,2]. It is reported that the deposition of Aβ and phosphorylated tau induces inflammation in the brain, thus exacerbating cognitive decline [3,4,5]. Microglia modulate the inflammation in the neuronal tissue. Infiltration and activation of microglia around senile plaques and NFTs are the prominent features of AD, and massive activation of microglia is associated with progression of the disease [4]. The suppression of microglial activation has been attracting attention as an approach for preventing AD [6,7].

Recent plural epidemiological studies have concluded that the consumption of dairy products reduces the risk of cognitive decline in the elderly and prevents AD [8]. It has been suggested that specific ingredients help promote healthy brain function during aging [9]. Ozawa et al. surveyed more than 1000 Japanese subjects aged 60–79 years and free from dementia to study their dietary patterns and whether these had any potential association with reduced risk of dementia [10,11]. They concluded that including milk or fermented dairy products in the diet reduced the risk of dementia in the general Japanese population. In a clinical trial, Ogata et al. [12] found an association between intake of dairy products and better short-term memory. They investigated sets of twins, comprising 78 men and 278 women, using the logical memory I (LM-I) of the revised Wechsler memory scale. Their findings revealed a significant association between dairy product intake and the LM-I scores in men. It also demonstrated that supplementation with whey peptide including GTWY peptide (β-lactolin) improved cognitive function in 114 elderly men and women. In this investigation, the visual paired-associates I of the revised Wechsler memory scale were improved in the whey peptide group [13]. These reports suggest that some peptides in dairy products are beneficial for the prevention of cognitive decline.

We demonstrated previously that intake of a dairy product fermented with *Penicillium candidum*, i.e., Camembert cheese, suppressed Aβ deposition and microglial activation in the brains of AD model (5×FAD) mice [14]. The microglial activation suppressing activity was induced by the process of fermentation with fungi, which suggests that the active ingredients are peptides. In addition, we identified dipeptides of tryptophan–tyrosine (WY) and tryptophan–methionine (WM) that suppressed the activation of primary microglia from the casein enzymatic digestion. Furthermore, we showed that consumption of WY peptide suppressed inflammation in the brain and ameliorated cognitive decline in 5×FAD mice [15]. However, the effects of WM peptide on inflammation in the brain and the pathology of AD have not been investigated. The present study evaluates the effects of WM peptide on brain inflammation using AD model (5×FAD) mice.

## 2. Results

### 2.1. Effects of WM Peptide on Aβ Deposition in 5×FAD Mice

To assess the effects of WM peptide on Aβ deposition in the brain, WM peptide was fed to 5×FAD mice, and the levels of Aβ were measured using immunohistochemistry and ELISA.

**Figure 1:**
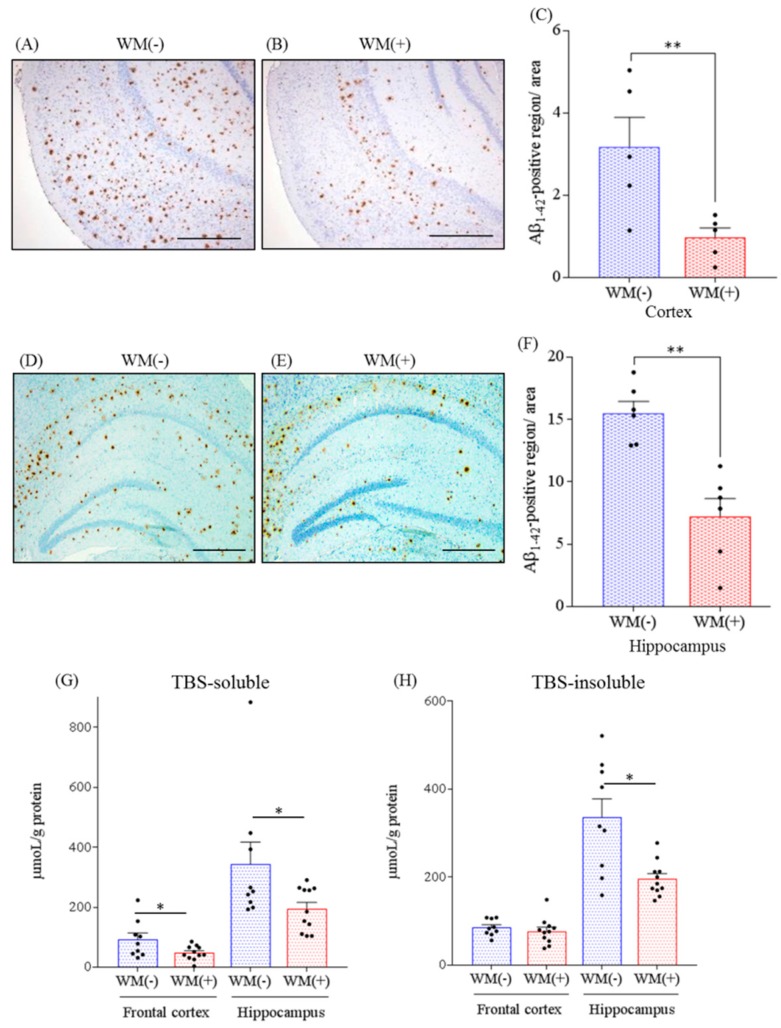
Effects of WM peptide on β amyloid (Aβ) deposition in 5×FAD mice. For 3 months, 2.5-month-old transgenic 5×FAD and wild-type female mice were fed a diet either containing or not containing 0.05% w/w WM peptide. Percentage of Aβ_1–42_ positive area was detected by immunohistochemistry in the brains of the transgenic control mice [WM(−)] and transgenic mice fed on a diet containing WM peptide [WM(+)]. (**A**), (**B**), (**D**) and (**E**): The representative immunohistochemistry images including olfactory-entorhinal cortex and hippocampus for Aβ_1–42_ in transgenic mice with or without WM peptide, respectively. (**C**) and (**F**): Semiquantification for Aβ_1–42_ detected immunohistochemically in cortex and hippocampus, respectively. (**G**) and (**H**): The levels of Tris-buffered saline (TBS)-soluble Aβ_1–42_ and TBS-insoluble and TBS-T-soluble Aβ_1–42_, respectively, in the frontal cortex and hippocampus were measured by ELISA. Scale bars indicate 500 μm (A and B)and 400 μm (D and E), respectively. Data represent the mean ± SEM values. There were 5–7 mice in each group for immunohistochemistry and 9 control transgenic mice and 11 transgenic mice were fed on a diet containing the WM peptide for ELISA. The *p*-values shown in the graph were calculated via the Student’s *t*-test. * *p* < 0.05 and ** *p* < 0.01.

The immunohistochemistry measurements showed that the levels of Aβ_1–42_ in the cortex (Figure 1A,B) and hippocampus (Figure 1D,E) were significantly lower in the group fed with WM peptide than they were in the control group (Figure 1C,F). Quantification by ELISA revealed that the levels of Tris-buffered saline (TBS)-soluble Aβ_1–42_ in the hippocampus and frontal cortex in the group fed with WM peptide were significantly lower than were those in the control group (Figure 1G). The levels of TBS-insoluble and TBS-T-soluble Aβ_1–42_ in the hippocampus in the group fed with WM peptide were significantly lower than were those in the control group (Figure 1H). These findings suggest that consumption of WM peptide reduced the deposition of Aβ_1–42_ in both the cortex and hippocampus.

### 2.2. Effects of WM Peptide on Inflammation and Microglial Activation in 5×FAD Mice

To evaluate the effects of WM peptide on inflammation, the levels of cytokines and chemokine in the hippocampus were measured. The levels of IL-1β, TNF-α, IL-6, IL-12p40, IL-12p70, and MIP-1α were increased significantly in the control 5×FAD mice compared with the wild-type mice (Table 1). The levels of IL-1β, TNF-α, IL-6 and MIP-1α were reduced significantly in the 5×FAD mice fed with WM peptide compared with the control 5×FAD mice.

**Figure 2:**
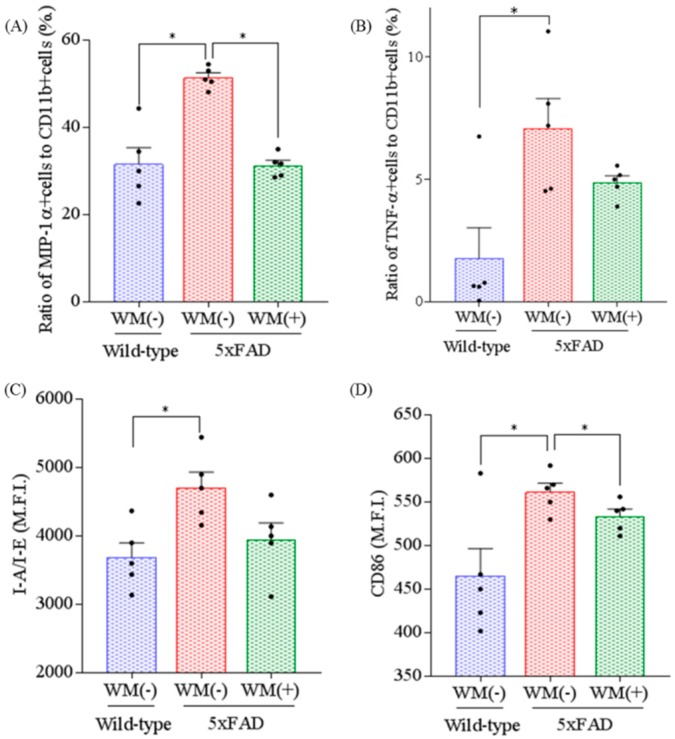
Effects of WM peptide on the activation of microglia in 5×FAD mice. For 3 months, 2.5-month-old transgenic 5×FAD and wild-type female mice were fed a diet either containing or not containing 0.05% *w*/*w* WM peptide. Characterization of CD11b-positive microglia in the brain isolated with magnetic cell sorting was performed by flow cytometry. (**A**) and (**B**), The ratios of MIP-1α and TNF-α-producing cells to CD11b-positive cells, respectively. (**C**) and (**D**), The expressions of I-A/I-E and CD86 on CD11b-positive cells, respectively. M.F.I. is the mean fluorescent intensity. Data represent the mean ± SEM values of 5 mice per group. *p*-values shown in the graph were calculated by one-way ANOVA, followed by the Tukey–Kramer test. * *p* < 0.05.

To evaluate the effects of WM peptide on microglial activation, microglia in the brain were characterized using a flow cytometer. The ratio of MIP-1α and TNF-α-producing cells to CD11b-positive cells and the expression of I-A/I-E and CD86 in CD11b-positive cells were increased significantly in the control 5×FAD mice compared with the wild-type mice (Figure 2A–D, respectively). The ratio of MIP-1α-producing cells and the expression of CD86 were significantly lower in the 5×FAD mice fed with WM peptide compared with the control 5×FAD mice (Figure 2A,D, respectively).

**Figure 3:**
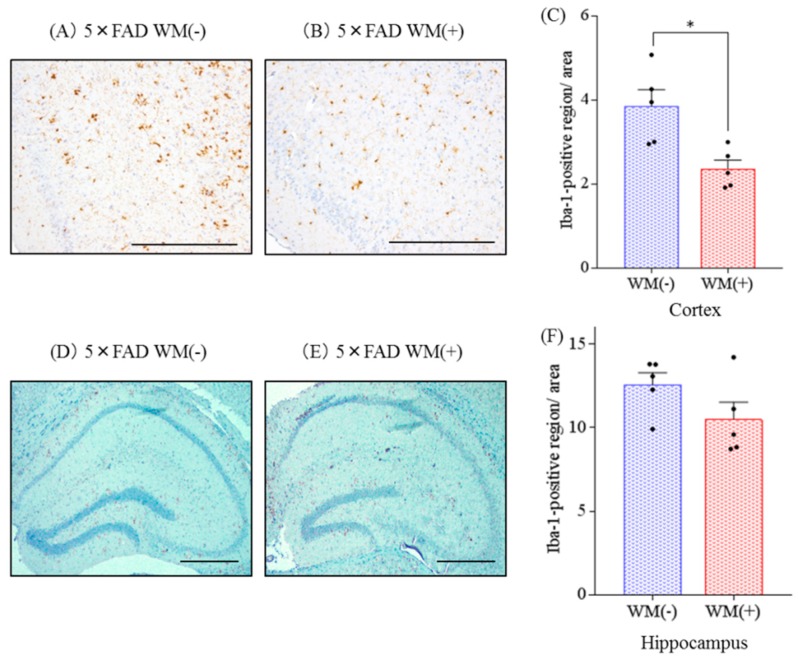
Effects of WM peptide on infiltration of microglia in 5×FAD mice. Percent positive area for Iba-1 in the brain of the transgenic control mice and transgenic mice fed on a diet containing WM peptide. (**A**), (**B**), (**D**) and (**E**), The representative immunohistochemistry images including olfactory cortex and hippocampus for Iba-1 in transgenic mice with or without WM peptide, respectively. (**C**) and (**F**), Semiquantification for Iba-1 detected immunohistochemically in each brain cortex and hippocampus, respectively. Scale bars indicate 100μm (A and B)and 400 μm (D and E), respectively. Data represent the mean ± SEM values of 5–6 mice per group. *p*-values shown in the graph were calculated via the Student’s *t*-test. * *p* < 0.05.

Additionally, the distribution of activated microglia in the brain was observed immunohistochemically (Figure 3A,B). The levels of Iba-1 positive microglia in the cortex were reduced significantly in the 5×FAD mice fed with WM peptide compared with the control 5×FAD mice (Figure 3C). These changes were not observed in the hippocampus (Figure 3D–F).

These findings suggest that consumption of WM suppressed the inflammation and microglial activation induced by Aβ deposition in 5×FAD mice

### 2.3. Effects of WM Peptide on Memory Impairment in 5×FAD Mice

**Figure 4:**
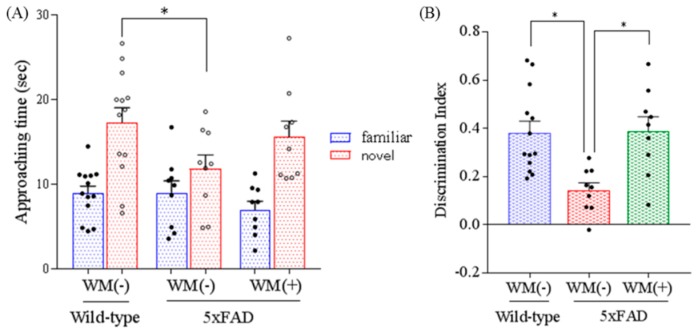
Effects of WM peptide on object recognition memory in 5×FAD mice. For 3 months, 2.5-month-old transgenic 5×FAD and wild-type female mice were fed a diet either containing or not containing 0.05% *w*/*w* WM peptide. Mice underwent a novel object recognition test. (**A**) and (**B**): Time which mice approach either familiar or novel object, respectively, and the discrimination index in the retention step during 5 min. Data represent the mean ± SEM values of 13 wild-type mice, 9 control transgenic mice, and 9 transgenic mice fed on a diet containing the WM peptide. *p*-values shown in the graph were calculated by one-way ANOVA followed by the Tukey–Kramer test. * *p* < 0.05.

To evaluate the object memory function, 5×FAD mice underwent a novel object recognition test. The time approaching novel object and discrimination index were reduced significantly in the control 5×FAD mice compared with the wild-type mice (Figure 4A). The discrimination index in the 5×FAD mice fed with WM peptide was increased significantly compared with that in the control 5×FAD mice (Figure 4B). These findings suggest that consumption of WM ameliorated long-term object memory impairment in 5×FAD mice.

## 3. Discussion

The present study shows that, in 5×FAD AD model mice, consumption of WM peptide reduced Aβ deposition and inflammation in the hippocampus and cortex and suppressed microglial activation. Consumption of WM peptide also ameliorated memory impairment in 5×FAD mice. These findings indicate that consumption of WM peptide prevents AD pathology by regulating microglial activation.

Previously, we identified WY peptide and WM peptide from β-casein as responsible agents for suppressing TNF-α production and increasing Aβ phagocytosis in an in vitro assay using primary microglia [15]. Administration of WY peptide reduced Aβ deposition and inflammation in the brain and suppressed microglial activation in lipopolysaccharide (LPS)-inoculated mice and 5×FAD mice [15]. However, the effects of WM peptide in vivo have not been investigated, and this is the first demonstration of the effects of WM peptide on Alzheimer’s pathology.

A previous study showed that dipeptides that include tryptophan in the N-terminal have the highest affinity for peptide transporters [16] and that a WY peptide labeled with a radioisotope was delivered directly to the brain. Single administration of dipeptides that include tryptophan in the N-terminal ameliorated memory impairment by modulating the dopamine system [17]. It is suggested that WM peptide was absorbed smoothly into the body and delivered to the brain to suppress the activity of microglia. It has been reported recently that orally administered WY peptide inhibits the activity of monoamine oxidase B (MAO-B) in the brain, indicating that dipeptides with tryptophan in the N-terminal, such as WM peptide, modulate the dopamine system [18]. It has also been reported that the MAO-B inhibitor reduces reactive oxygen species (ROS) and suppresses the pathology of Parkinson’s disease [19]. In addition, it has been shown that danshensu, an active ingredient of *Salvia miltiorrhiza* that inhibits the activity of MAO-B, reduces the activation of NF-κB and suppresses the NF-κB-regulated pro-inflammatory responses [20]. It has been reported that brain inflammation impaired cognitive function, including objective memory, and suppression of the inflammation attenuated the memory impairment [21,22]. Taking these findings together, it is suggested that, after oral administration, WM peptide is delivered to the brain where it inhibits MAO-B activity, resulting in the suppression of inflammatory responses in the brain. However, as this is speculation based on the findings of previous reports, we need to evaluate the MAO-B inhibitory activity and underlying mechanism of WM peptide in further studies.

Microglia play a central role in inflammation in the brain, and microglial activation is associated with the pathology of AD. On the one hand, microglia keep the environment free of waste products, including Aβ by phagocytosis. On the other hand, microglia infiltrate Aβ plaques in the brain, and as the disease pathology progresses, become massively activated, producing inflammatory cytokines and ROS, which exacerbates the pathology of AD. Inflammation in the brain has been reported to accelerate the cognitive decline and pathology of dementia. In addition, it has been reported that subjects with mild cognitive impairment show brain inflammation accompanies Aβ [23], so prevention of inflammation in the brain is a therapeutic and preventive target for dementia and other neurological disorders [24,25]. Suppression of inflammation in the brain by WM peptide might be associated with memory improvement.

Non-steroidal inflammatory drugs are being evaluated for prevention of AD [24,25]; however, medicinal treatments have a risk of side/adverse effects. A recent study has shown that bitter acids derived from hops prevent inflammation in the hippocampus and the frontal cortex that causes memory impairment in 5×FAD mice, tauopathy mice, and aged mice [26,27,28,29]. This safe and easy approach to tackling AD has been receiving increasing attention. WM peptide also suppresses the activation of microglia and prevents cognitive decline, so it might be considered as a potential new approach for preventing dementia.

The present study has some limitations. WM peptide is involved in cells other than microglia, including neurons and astrocytes, and cleavage of the Aβ precursor protein (APP) and the expression of APP were not evaluated [30]. Moreover, the underlying mechanism of the anti-inflammatory effects of WM peptide needs to be investigated in further studies. In addition, the present study used only female mice and did not evaluate wild-type mice with WM peptide, so we need to evaluate the effects of WM peptide on male 5×FAD and wild-type mice.

The present research is based on epidemiological and clinical studies that suggest that the intake of dairy products prevents cognitive decline and dementia [8]. In addition, the study elucidates the preventive effects of WM peptide on AD pathology. WM peptide is contained in various fermented dairy products, including blue cheese and chevre cheese (data not shown). These dairy products and other food materials rich in WM peptide are easy and safe to ingest in daily life, and they might represent new approaches for the prevention or amelioration of memory impairment related to neuronal inflammation.

## 4. Materials and Methods

### 4.1. Animals

AD model mice, B6SJL-Tg mice (APPSwFlLon, PSEN1*M146L*L286V, http://jaxmice.jax.org/strain/006554.html, [31]), hereafter referred to as 5×FAD transgenic mice, were purchased from Jackson Laboratory (Sacramento, CA, USA) and maintained by crossing hemizygous transgenic mice with B6SJLF1/J mice in the experimental facility at the University of Tokyo. The 5×FAD transgenic mice overexpress mutant human APP (695) with the Swedish (K670N, M671L), Florida (I716V), and London (V717I) familial AD (FAD) mutations, along with human PS1 harboring two FAD mutations, M146L and L286V. Non-transgenic wild-type littermates were used in all experiments. All experiments were approved by the Animal Care and Use Committee of the Graduate School of Agricultural and Life Sciences, the University of Tokyo, and were conducted from December 2015 to November 2016 in strict accordance with their guidelines (Approval ID; P15-042). All possible efforts were made to minimize suffering. Mice were maintained at room temperature (23 ± 1 °C) under constant 12 h light/dark cycles (light period from 8:00 am to 8:00 pm). Mice under 3 months of age were fed with a standard purified rodent growth diet (AIN-93G, Oriental Yeast, Tokyo, Japan), and those over 3 months were fed with a maintenance diet (AIN-93M, Oriental Yeast).

Mice were allocated into each group without significant differences about weight among the groups. To evaluate the effects of WM peptide on Alzheimer’s-like disease, 2.5-month-old transgenic 5×FAD and wild-type female mice were fed a diet that either contained or did not contain 0.05% *w*/*w* WM peptide (purity >97%, Bachem, Bubendorf, Switzerland) for 3 months. Before behavioral evaluation, mice were moved into a sound isolated room 16 h before the test started, and behavioral evaluations were carried out during the light phase. Data analysis was conducted by the performer of behavioral evaluation. After behavioral evaluation, the mice were euthanized and their brains were removed, as described in the following sections.

### 4.2. Quantification of Cytokine and Aβ by ELISA

Homogenate samples of the left hippocampus and the frontal cortex were prepared as described in a previous report [26]. Each sample of the hippocampus and frontal cortex was homogenized in a TBS buffer containing a protease inhibitor cocktail (BioVision, Milpitas, CA, USA) and using a multi-beads shocker (Yasui Kikai, Osaka, Japan). The pellets were homogenized again in TBS containing 1% Triton X-100 (Wako, Osaka, Japan), and the supernatant was collected after centrifugation at 50,000× *g* for 20 min. The total protein concentration of each supernatant was measured using the BCA Protein Assay Kit (Thermo-Scientific, Yokohama, Japan). The first supernatant was assayed to quantify soluble Aβ_1-42_ (Wako) by ELISA and cytokines and chemokines by a Bio-Plex assay system (Bio-Rad, Hercules, CA, USA). The second supernatant was used to quantify insoluble Aβ_1-42_ (Wako) by ELISA.

### 4.3. Immunohistochemistry

To evaluate Aβ deposition and microglial infiltration immunohistochemically, the right-brain hemispheres (*n* = 6–7 in each group) were fixed in 10% formalin solution (Wako), paraffin-embedded, and cut into 5 µm sections. Each coronal brain region included the hippocampus and cerebral cortex (bregma 2.30 mm posterior). Then, after being dewaxed and rehydrated, the sections for Aβ were treated in 98% formic acid and the sections for Iba-1 were autoclaved at 121 °C for 10 min in 0.2% citrate buffer (pH 6.0) for antigen retrieval. Then, the sections were treated with blocking solution (8% *w*/*v* skimmed milk) for 30 min after inactivation of endogenous peroxidase with 3% H_2_O_2_ (Wako) in methanol for 5 min. The sections were then incubated overnight at room temperature with either a monoclonal anti-human Aβ_x–42_ antibody (12F4, Millipore, Billerica, MA, USA) or a polyclonal anti-Iba-1 antibody (Wako) as primary antibodies. After being reacted with horseradish peroxidase coupled goat anti-mouse or rabbit IgG antibodies (4 μg/mL, Nichirei, Tokyo, Japan) for 1 h at room temperature, the sections were visualized with 3,3′-diaminobenzidine (Wako) and counterstained in hematoxylin. The size of the positive region per area (one section per each mouse) was measured using the Image J image analysis software (NIH, Bethesda, MD, USA).

### 4.4. Characterization of Microglia Using Flow Cytometry

To evaluate microglia activity, the right-brain hemispheres (*n* = 5 in each group) were removed, and isolated. CD11b-positive microglia were used for flow cytometry analysis, as described in a previous study [14,26,27]. Brain cells were obtained from mice by papain treatment using a Neural Tissue Dissociation Kit (P) (Miltenyi Biotec, Boston, MA, USA). The cells were treated with 2 μg/mL of anti-CD11b antibody conjugated with microbeads (Miltenyi Biotec), and CD11b-positive cells were isolated by magnetic cell sorting (MACS).

To measure the expression of cell surface markers, isolated microglia with more than 90% purity were stained with the following antibodies: anti-CD11b-APC-Cy7 (M1/70, BD Pharmingen, Sand Diego, CA, USA), anti-I-A/I-E-FITC (M5/114.15.2, eBioscience, San Diego, CA, USA), and anti-CD86-PE (GL-1, eBioscience), and were then analyzed using a FACS Canto II flow cytometer (BD Bioscience).

To measure intracellular cytokine production, isolated microglia were plated in a 96-well plate (BD Biosciences, San Diego, MA, USA) at 50,000 per well and cultured in DMEM/F-12 (Gibco, Carlsbad, CA, USA) medium supplemented with 10% fetal calf serum (Gibco) and 100 U/mL of penicillium/streptomycin (Sigma-Aldrich, St. Louis, MO, USA). Microglia were treated with a leukocyte activation cocktail using BD GolgiPlug (BD Biosciences) for 12 h and with a BD Cytofix/Cytoperm Fixation/Permeabilization kit (BD Biosciences), and were then stained with the following antibodies: anti-MIP-1α-PE (DNT3CC, eBiosciences), anti-TNF-α-FITC (MP6-XT22, BD Pharmingen), and anti-CD11b-APC-Cy7 (M1/70, BD Pharmingen). The cells were analyzed using a flow cytometer.

### 4.5. Novel Object Recognition Test

To evaluate memory function, a novel object recognition test was conducted during the light cycle in a polyvinyl chloride box (40 × 40 × 40 cm^3^) without a roof, in accordance with a previous demonstration [26,32]. This apparatus was placed in a sound-isolated experimental room, and was observed by means of a digital video camera mounted on the ceiling. Mice were moved into this room at least 12 h before the experiment was to begin. A pair of wooden triangle poles (4.5 × 4.5 × 4.5 cm^3^) or wooden pyramids (4.5 × 4.5 × 4.5 cm^3^) were used for the acquisition trial, and a pair of poles or pyramids and a golf ball (4.5 cm diameter) were used for the retention trial. In all trials, the objects were placed 7.5 cm from the corner of the box. In the acquisition trial, each mouse was allowed to explore the box containing the two objects for 10 min. Twenty-four hours after the acquisition trial, the mouse was allowed to explore the box containing both the novel and the familiar objects for 5 min. The discrimination index was calculated by dividing the difference in the time spent exploring the novel object and the familiar object by the total time spent exploring both objects: i.e., (novel object exploration time – familiar object exploration time)/(total exploration time). Thus, a discrimination index of 0 indicated equal exploration of both objects.

### 4.6. Statistical Analysis

The data represent the mean ± SEM values. Data were analyzed using one-way ANOVA followed by either the Tukey–Kramer test or Student’s *t*-test, as described in the figure legends. A *p*-value of <0.05 was considered statistically significant. All statistical analyses were performed using the Ekuseru–Toukei 2012 software program (Social Survey Research Information, Tokyo, Japan).

## Figures and Tables

**Table 1:** Cytokine and chemokine levels in the hippocampus.

Item	Wild-Type	5×FAD	
	WM(−)	WM(−)	WM(+)
IL-1β	6.55 ± 0.62	12.06 ± 0.92 *	8.88 ± 1.07 ^†^
TNF-α	10.02 ± 1.78	14.72 ± 0.84 *	11.30 ± 1.04 ^†^
IL-6	0.27 ± 0.03	0.35 ± 0.04 *	0.23 ± 0.06 ^†^
IL-12p40	2.06 ± 0.23	2.49 ± 0.12 *	2.16 ± 0.32
IL-12p70	1.09 ± 0.19	1.32 ± 0.09 *	1.18 ± 0.22
MIP-1α	3.99 ± 1.26	35.87 ± 4.16 **	22.67 ± 3.47 ^††^

For 3 months, 2.5-month-old transgenic 5×FAD and wild-type female mice were fed a diet either containing or not containing 0.05% *w*/*w* WM peptide. The levels of cytokines and chemokines in the hippocampus were measured by ELISA. Data represent the mean ± SEM values of 15 wild-type mice, 11 control transgenic mice, and 12 transgenic mice fed on a diet containing the WM peptide. *p*-values shown in the graph were calculated by one-way ANOVA followed by the Tukey–Kramer test. *, † *p* < 0.05 and **, †† *p* < 0.01.

## Abbreviations

Aβ	amyloid beta
AD	Alzheimer’s disease
ELISA	enzyme-linked immunosorbent assay
FAD	familial Alzheimer’s disease
IL	Interleukin
LM-1	logical memory 1
LPS	lipopolysaccharide
MACS	magnetic cell sorting
MIP-1α	macrophage inflammatory protein-1α
NFT	neurofibrillary tangle
TBS	Tris-buffered saline
TNF-α	tumor necrosis factor α
WM	Tryptophan–methionine
WY	Tryptophan–tyrosine
